# First Complete Genome Sequence of a Simian Foamy Virus Infecting the Neotropical Primate Brachyteles arachnoides

**DOI:** 10.1128/MRA.00839-18

**Published:** 2018-07-19

**Authors:** Cláudia P. Muniz, Liliane T. F. Cavalcante, Dawn M. Dudley, Alcides Pissinatti, David H. O’Connor, André F. Santos, Marcelo A. Soares

**Affiliations:** a Programa de Oncovirologia, Instituto Nacional de Câncer, Rio de Janeiro, Brazil; b Departamento de Genética, Universidade Federal do Rio de Janeiro, Rio de Janeiro, Brazil; c Department of Pathology and Laboratory Medicine, University of Wisconsin—Madison, School of Medicine and Public Health, Madison, Wisconsin, USA; d Centro de Primatologia do Rio de Janeiro, Rio de Janeiro, Brazil; e Wisconsin National Primate Research Center, University of Wisconsin—Madison, Madison, Wisconsin, USA; Queens College

## Abstract

The complete genome sequence of a simian foamy virus infecting the neotropical primate Brachyteles arachnoides (SFVbar) was obtained using next-generation sequencing and genome walking. The full-length SFVbar genome is composed of 11,994 bp and shows a genomic organization similar to that of other neotropical SFVs.

## ANNOUNCEMENT

Simian foamy viruses (SFV) naturally infect a wide diversity of nonhuman primates, including Old World primates (OWP) (1–3) and New World primates (NWP) (4–7) and can be transmitted to exposed humans, mainly through contact with saliva (8–15). Although no disease has been associated with SFV infection in natural hosts (16), a study of SFV-infected Cameroonian hunters evidenced mild to moderate hematological abnormalities (17). Humans exposed to NWP SFV were found positive by serological assays, but no viral sequence was detected (6, 18). While SFV was detected in a wide diversity of NWP (4, 5, 7), only four complete genome sequences of SFV from NWP are available (19–21). Novel NWP SFV sequences are useful for developing screening assays to study SFV infection in humans.

A saliva sample was collected from a Brachyteles arachnoides hosted at the Primate Center of Rio de Janeiro by following the national guidelines and provisions of Instituto Brasileiro do Meio Ambiente e dos Recursos Naturais Renováveis, Brazil, under permanent license number 11375-1, which included animal welfare standard operating procedures. The project was approved by the Ethics Committee on the Use of Animals of Universidade Federal do Rio de Janeiro (037/14). Nucleic acids (both DNA and RNA) were extracted from saliva using a QIAamp MinElute virus spin kit. Following reverse transcription-PCR (RT-PCR) and library preparation using the Nextera XT DNA library preparation kit (Illumina), deep sequencing was conducted using the Illumina MiSeq V2 600-cycle kit. FASTq reads were loaded in DNAnexus, and after removal of human and known microbial contaminant reads with the viral-ngs-human-depletion tool from the Broad Institute and trimming of low-quality reads using bbmap (Joint Genome Institute), *de novo* assembly was carried out using MEGAHIT version 0.3.3. Four contigs of 595, 826, 543, and 548 bp comprising 21 reads with lengths of >150 nucleotides (nt) mapped to a spider monkey foamy virus sequence (GenBank accession number EU010385). These contigs were used to design specific primers, and genome sequence walking was performed with Sanger sequencing to obtain the virus’ complete genome using genomic DNA from saliva.

An 11,994-bp full-length genome of SFV infecting B. arachnoides was assembled. The virus was named SFVbar in accordance with the nomenclature recently published (22). The GenBank ORFfinder tool was used to determine the location and size of SFVbar open reading frames (ORFs). The genomic structure of SFVbar was similar to that of other foamy viruses (FV) (23), with ORFs encoding the essential proteins Gag, Pol, and Env and the accessory proteins Tas and Bet, flanked by two long terminal repeats. Additionally, the SFVbar sequence was aligned with the four NWP SFV genome sequences available from GenBank (SFVsxa, accession number KP143760; SFVcja, accession number GU356395; SFVasp, accession number EU010385; and SFVspp, accession number GU356394) using MUSCLE in MEGA version 7.021. A phylogenetic tree inferred using the neighbor-joining method, the Kimura 2-p model, and 1,000 bootstrap replicates grouped SFVbar with SFVasp, both infecting NWP species of the Atelidae family. These findings corroborate the FV-host cospeciation hypothesis (24). A nucleotide sequence comparison using the pairwise distance tool of MEGA showed that SFVbar is more similar to SFVasp in the *pol* gene (75.7% sequence identity) but less in *env* (68.8%) and in *gag* (55.8%).

### Data availability.

The SFVbar genome sequence was deposited in DDBJ/ENA/GenBank under accession number MH368762.

## References

[1] Huang F, Wang H, Jing S, Zeng W 2012 Simian foamy virus prevalence in *Macaca mulatta* and zookeepers. AIDS Res Hum Retroviruses 28:591–593. doi:10.1089/aid.2011.0305. 22236106

[2] Morozov VA, Leendertz FH, Junglen S, Boesch C, Pauli G, Ellerbrok H 2009 Frequent foamy virus infection in free-living chimpanzees of the Taï National Park (Côte d'Ivoire). J Gen Virol 90:500–506. doi:10.1099/vir.0.003939-0. 19141461

[3] Calattini S, Nerrienet E, Mauclère P, Georges-Courbot M-C, Saïb A, Gessain A 2004 Natural simian foamy virus infection in wild-caught gorillas, mandrills and drills from Cameroon and Gabon. J Gen Virol 85:3313–3317. doi:10.1099/vir.0.80241-0. 15483245

[4] Muniz CP, Troncoso LL, Moreira MA, Soares EA, Pissinatti A, Bonvicino CR, Seuánez HN, Sharma B, Jia H, Shankar A, Switzer WM, Santos AF, Soares MA 2013 Identification and characterization of highly divergent simian foamy viruses in a wide range of New World primates from Brazil. PLoS One 8:e67568. doi:10.1371/journal.pone.0067568. 23844033 PMC3701081

[5] Muniz CP, Jia H, Shankar A, Troncoso LL, Augusto AM, Farias E, Pissinatti A, Fedullo LP, Santos AF, Soares MA, Switzer WM 2015 An expanded search for simian foamy viruses (SFV) in Brazilian New World primates identifies novel SFV lineages and host age-related infections. Retrovirology 12:94. doi:10.1186/s12977-015-0217-x. 26576961 PMC4650395

[6] Muniz CP, Cavalcante LTF, Jia H, Zheng H, Tang S, Augusto AM, Pissinatti A, Fedullo LP, Santos AF, Soares MA, Switzer WM 2017 A non-invasive specimen collection method and a novel simian foamy virus (SFV) DNA quantification assay in New World primates reveal aspects of tissue tropism and improved SFV detection. PLoS One 12:e0184251 https://www.ncbi.nlm.nih.gov/pubmed/28863180. 28863180 10.1371/journal.pone.0184251 PMC5581185

[7] Ghersi BM, Jia H, Aiewsakun P, Katzourakis A, Mendoza P, Bausch DG, Kasper MR, Montgomery JM, Switzer WM 2015 Wide distribution and ancient evolutionary history of simian foamy viruses in New World primates. Retrovirology 12:89. doi:10.1186/s12977-015-0214-0. 26514626 PMC4627628

[8] Rua R, Betsem E, Gessain A 2013 Viral latency in blood and saliva of simian foamy virus-infected humans. PLoS One 8:e77072. doi:10.1371/journal.pone.0077072. 24116202 PMC3792900

[9] Mouinga-Ondémé A, Kazanji M 2013 Simian foamy virus in non-human primates and cross-species transmission to humans in Gabon: an emerging zoonotic disease in Central Africa? Viruses 5:1536–1552. doi:10.3390/v5061536. 23783811 PMC3717720

[10] Mouinga-Ondémé A, Caron M, Nkoghé D, Telfer P, Marx P, Saib A, Leroy E, Gonzalez J-P, Gessain A, Kazanji M 2012 Cross-species transmission of simian foamy virus to humans in rural Gabon, Central Africa. J Virol 86:1255–1260. doi:10.1128/JVI.06016-11. 22072747 PMC3255803

[11] Betsem E, Rua R, Tortevoye P, Froment A, Gessain A 2011 Frequent and recent human acquisition of simian foamy viruses through apes’ bites in Central Africa. PLoS Pathog 7:e1002306. doi:10.1371/journal.ppat.1002306. 22046126 PMC3203161

[12] Mouinga-Ondémé A, Betsem E, Caron M, Makuwa M, Sallé B, Renault N, Saib A, Telfer P, Marx P, Gessain A, Kazanji M 2010 Two distinct variants of simian foamy virus in naturally infected mandrills (*Mandrillus sphinx*) and cross-species transmission to humans. Retrovirology 7:105. doi:10.1186/1742-4690-7-105. 21156043 PMC3009703

[13] Khan AS 2009 Simian foamy virus infection in humans: prevalence and management. Expert Rev Anti Infect Ther 7:569–580. doi:10.1586/eri.09.39. 19485797

[14] Calattini S, Betsem EBA, Froment A, Mauclère P, Tortevoye P, Schmitt C, Njouom R, Saib A, Gessain A 2007 Simian foamy virus transmission from apes to humans, rural Cameroon. Emerg Infect Dis 13:1314–1320. doi:10.3201/eid1309.061162. 18252101 PMC2857270

[15] Switzer WM, Bhullar V, Shanmugam V, Cong M, Parekh B, Lerche NW, Yee JL, Ely JJ, Boneva R, Chapman LE, Folks TM, Heneine W 2004 Frequent simian foamy virus infection in persons occupationally exposed to nonhuman primates. J Virol 78:2780–2789. doi:10.1128/JVI.78.6.2780-2789.2004. 14990698 PMC353775

[16] Pinto-Santini DM, Stenbak CR, Linial ML 2017 Foamy virus zoonotic infections. Retrovirology 14:55. doi:10.1186/s12977-017-0379-9. 29197389 PMC5712078

[17] Buseyne F, Betsem E, Montange T, Njouom R, Bilounga Ndongo C, Hermine O, Gessain A 2018 Clinical signs and blood-test results of humans infected with zoonotic simian foamy viruses: a case-control study. J Infect Dis 218:144–151. doi:10.1093/infdis/jiy181. 29608711

[18] Stenbak CR, Craig KL, Ivanov SB, Wang X, Soliven KC, Jackson DL, Gutierrez GA, Engel G, Jones-Engel L, Linial ML 2014 New World simian foamy virus infections *in vivo* and *in vitro*. J Virol 88:982–991. doi:10.1128/JVI.03154-13. 24198412 PMC3911628

[19] Pacheco B, Finzi A, McGee-Estrada K, Sodroski J 2010 Species-specific inhibition of foamy viruses from South American monkeys by New World Monkey TRIM5α proteins. J Virol 84:4095–4099. doi:10.1128/JVI.02631-09. 20130055 PMC2849481

[20] Thümer L, Rethwilm A, Holmes EC, Bodem J 2007 The complete nucleotide sequence of a New World simian foamy virus. Virology 369:191–197. doi:10.1016/j.virol.2007.07.018. 17765280

[21] Troncoso LL, Muniz CP, Siqueira JD, Curty G, Schrago CG, Augusto A, Fedullo L, Soares MA, Santos AF 2015 Characterization and comparative analysis of a simian foamy virus complete genome isolated from Brazilian capuchin monkeys. Virus Res 208:1–6. doi:10.1016/j.virusres.2015.05.022. 26047587

[22] Khan AS, Bodem J, Buseyne F, Gessain A, Johnson W, Kuhn JH, Kuzmak J, Lindemann D, Linial ML, Löchelt M, Materniak-Kornas M, Soares MA, Switzer WM 2018 Spumaretroviruses: updated taxonomy and nomenclature. Virology 516:158–164. doi:10.1016/j.virol.2017.12.035. 29407373 PMC11318574

[23] Rethwilm A 2010 Molecular biology of foamy viruses. Med Microbiol Immunol 199:197–207. doi:10.1007/s00430-010-0158-x. 20445989

[24] Switzer WM, Salemi M, Shanmugam V, Gao F, Cong M, Kuiken C, Bhullar V, Beer BE, Vallet D, Gautier-Hion A, Tooze Z, Villinger F, Holmes EC, Heneine W 2005 Ancient co-speciation of simian foamy viruses and primates. Nature 434:376–380. doi:10.1038/nature03341. 15772660

